# HBO Alleviates Neural Stem Cell Pyroptosis via lncRNA-H19/miR-423-5p/NLRP3 Axis and Improves Neurogenesis after Oxygen Glucose Deprivation

**DOI:** 10.1155/2022/9030771

**Published:** 2022-02-07

**Authors:** Yuqin Ye, Zhusheng Feng, Shilai Tian, Yongxiang Yang, Yibin Jia, Guanyi Wang, Jiayou Wang, Wei Bai, Jinsheng Li, Xiaosheng He

**Affiliations:** ^1^Department of Neurosurgery, Xijing Hospital, Air Force Medical University, Xi'an 710032, China; ^2^Department of Aerospace Medicine, Air Force Medical University, Xi'an 710032, China; ^3^Department of Neurosurgery, PLA 921st Hospital, Changsha 410000, China; ^4^Department of Emergency, Xijing Hospital, Air Force Medical University, Xi'an 710032, China; ^5^Department of Neurosurgery, The First Hospital of Lan Zhou University, Lan Zhou 730099, China; ^6^Department of Neurosurgery, The General Hospital of Western Theater Command, Chengdu 610083, China

## Abstract

Due to the limited neurogenesis capacity, there has been a big challenge in better recovery from neurological dysfunction caused by stroke for a long time. Neural stem cell (NSC) programmed death is one of the unfavorable factors for neural regeneration after stroke. The types of death such as apoptosis and necroptosis have been deeply investigated while the pyroptosis of NSCs is not quite understood. Although it is well accepted that hyperbaric oxygen (HBO) alleviates the oxygen-glucose deprivation (OGD) injury after stroke and reduces programmed death of NSCs, whether NSC pyroptosis is involved in this process is still unknown. Therefore, this study is aimed at studying the potential effect of HBO treatment on NSC pyroptosis following OGD exposure, as well as its influence on NSC proliferation and differentiation *in vitro*. The results revealed that OGD increased NOD-like receptor protein 3 (NLRP3) expression to induce the pyroptotic death of NSCs, which was rescued by HBO treatment. And the upregulated lncRNA-H19 functioned as a molecular sponge of miR-423-5p to target NLRP3 for NSC pyroptosis following OGD. Most importantly, it was confirmed that HBO exerted protection of NSCs against pyroptosis by inhibiting lncRNA-H19/miR-423-5p/NLRP3 axis. Moreover, HBO restraint of lncRNA-H19-associated pyroptosis benefited the proliferation and neuronal differentiation of NSCs. It was concluded that HBO attenuated NSC pyroptosis via lncRNA-H19/miR-423-5p/NLRP3 axis and enhanced neurogenesis following OGD. The findings provide new insight into NSC programmed death and enlighten therapeutic strategy after stroke.

## 1. Introduction

Ischemic stroke is a leading cerebral vascular event associated with high morbidity, disability, and mortality in central nervous system. Neural stem cells (NSCs), located in the subgranular zone of adult hippocampus, can proliferate and differentiate into various kinds of cells in order to repair the injured brain [1]. It has been documented that ischemic injury activates hippocampal NSCs to generate newborn neurons for neurogenesis, whereas the amount is very limited and not enough to replenish the damaged cells for neurological function restoration [2]. One of the main reasons is that in the case of oxygen-glucose deprivation (OGD) after stroke, a host of NSC programmed deaths occur before neurogenesis begins. Although a lot of studies have elucidated the types of programmed death such as apoptosis and necroptosis in response to OGD exposure, little is known about NSC pyroptosis [3, 4].

Long noncoding RNA (lncRNA), a kind of noncoding RNA with more than 200 nucleotides, participates in various biological activities via diverse mechanisms such as epigenetic, transcriptional, and posttranscriptional modulation [5, 6]. Abundant studies revealed that lncRNA-H19, an imprinted gene seated at chromosome 11, exerted important roles on the onset and development of ischemic stroke, involving neuronal apoptosis and autophagy, microglial polarization, and inflammatory response [7–9]. Notably, the latest researches have provided much evidence that lncRNA-H19 is involved in microglia pyroptosis induced by retinal ischemia injury and cardiomyocyte pyroptosis triggered by myocardial ischemia infarction [10–12]. To the best of our knowledge, whether and how lncRNA-H19 contributes to NSC pyroptosis and affects neurogenesis following OGD has never been investigated. NOD-like receptor protein 3 (NLRP3) inflammasome assembly is essential for the downstream Caspase-1 activation and gasdermin D (GSDMD) pore formation in the canonical pyroptosis signaling [13, 14]. Online bioinformatic analysis and screening demonstrate that lncRNA-H19 is predicted to bind microRNA-423-5p (miR-423-5p) directly, and miR-423-5p is predicted to bind the 3′UTR of NLRP3. According to the molecular sponger mechanism of lncRNA [15], we hypothesize that OGD induces lncRNA-H19 competitively binding miR-423-5p to upregulate the expression of NLRP3, which initiates the pyroptotic death of NSCs and further affects their neurogenesis capacity.

Hyperbaric oxygen (HBO), a practical approach to ameliorate hypoxic state, has been shown to promote neurogenesis and facilitate neurological function recovery after stroke [13, 14]. HBO treatment not only enhances the viability and survival of NSCs but also enhances NSC proliferation and differentiation into neurons after ischemic injury [16–18]. Recent studies have proven that HBO exerts therapeutic roles in various pathophysiologies of hypoxic/ischemic diseases by regulating noncoding RNA [19–21]. In this present study, an OGD exposure and HBO treatment model of NSCs *in vitro* was established with a purpose to investigate the effect and mechanism of HBO treatment on the presumed lncRNA-H19-associated pyroptosis of NSCs following OGD and clarify its influence on NSC proliferation and differentiation.

## 2. Materials and Method

### 2.1. Reagents and Animals

Dulbecco's modified Eagle's medium/nutrient mixture F12 (DMEM/F12, 11330057), epidermal growth factor (EGF, PMG8043), basic fibroblast growth factor (bFGF, PMG0033), L-glutamine (25030081), and B27 serum-free supplement (17504044) were purchased from Thermo Fisher Scientific (MA, USA). Cell counting kits-8 (CCK-8, C0042), lactate dehydrogenase (LDH) measurement kits (C0017), bicinchoninic acid assay (BCA) kits (P0011), interleukin-1*β* (IL-1*β*, PI301), and interleukin-18 (IL-18, PI553) enzyme-linked immunosorbent assay (ELISA) kits were purchased from Beyotime Biotechnology (Shanghai, China). Radio immunoprecipitation assay (RIPA) lysis buffer (PR20001) was purchased from Proteintech (Wuhan, China). NLRP3 special agonist nigericin (Nig, 28380-24-7, administrated concentration 10 *μ*mol/L) and antagonist MCC950 (256373-96-3, administrated concentration 1 *μ*mol/L) were purchased from MedChemExpress (NJ, USA). C57BL/6 male mice were conducted under the laboratory animal care and use guidelines of Air Force Medical University. All efforts were made to reduce animal suffering.

### 2.2. NSC Culture

As described in the previous study [22], hippocampal tissues were dissected from the brain of 8-week-old C57BL/6 mice and digested by trypsin at 37°C for 30 min. After the samples were centrifuged and resuspended, NSCs were collected and cultured in DMEM/F12 medium supplemented with 20 *μ*l/ml B27, 2 mM L-glutamine, 20 ng/ml EGF, and 20 ng/ml bFGF at the density of 1 × 10^5^ cells/cm^2^. The culture medium was updated every three days, and cells passage started on the seventh day. NSCs of the third passage were planted at poly-L-ornithine/fibronectin precoated dishes for further experiments.

### 2.3. OGD Exposure and HBO Treatment

For OGD exposure, NSC culturing medium was replaced with glucose-free medium (Earle's balanced salt solution); then, the cells were transferred into an anaerobic chamber filled with 95% N2 and 5% CO_2_ for 6 h incubation at 37°C. Afterward, NSCs were put back to normal culturing medium and incubated in normal oxygen condition described above. For HBO treatment, the OGD-exposed NSCs were placed in a hyperbaric chamber (DWC450-1150, 701 Institute of Shipbuilding Industry Corporation, Shanghai, China) filled with 100% oxygen and subjected to HBO treatment according to the previous study [16]. Pressure in the chamber increased to a preset value in the initial 20 min and was maintained at this preset pressure during the next 60 min, and then, the pressure declined at a constant speed to normal pressure in the following 20 min. The whole process was performed under appropriate humidity and temperature, and oxygen concentration in the HBO chamber was not less than 90%. The preset pressure in different groups was, respectively, 1.0, 1.5, 2.0, and 2.5 atmospheres (ATA).

### 2.4. Cell Viability Assay

CCK-8 colorimetric assay was performed to detect NSC viability in different groups. Briefly, NSCs were plated into 96-well plates at the density of 5 × 10^3^ cells/well. After treatment in each group, 10 *μ*l CCK-8 solution was added to each well where NSCs were incubated at 37°C for 2 h. The absorbance was determined at 450 nm using a microplate reader (Bio-Rad, CA, USA). The examination was repeated three times, and blank controls were set at the same time.

### 2.5. LDH Measurement

NSCs were planted into plates in which the medium would be replaced with fresh serum-free culture medium when the density was 80%. Following treatment in each group, 20 *μ*l LDH assay reagent was added and the NSCs were incubated for 1 h. A total of 120 *μ*l supernatant in each well was collected for detecting the LDH released from NSCs in a microplate absorbance reader (Bio-Rad, CA, USA). The procedures were conducted according to the instructions from manufacturer.

### 2.6. ELISA

The inflammatory cytokines IL-1*β* and IL-18 released from NSCs were quantified by corresponding ELISA detection kits. NSC supernatants in different groups were collected and centrifuged for measurement in a microplate absorbance reader (Bio-Rad, CA, USA) at 450 nm. The assays were performed based on the instructions from manufacturer.

### 2.7. Western Blot (WB)

NSCs were digested in cold RIPA lysis buffer according to the instructions. Following lysate centrifugation and BCA assay, 40 *μ*g of protein was loaded by sodium dodecyl sulfate-polyacrylamide gel electrophoresis and transferred to polyvinylidene membranes. After being blocked with 5% milk without fat in tris-buffered saline with 0.1% Triton X-100 buffer, the membranes were probed with primary antibodies at 4°C overnight. Then, the membranes were incubated with horseradish peroxidase-conjugated secondary antibodies (1 : 20000, A0208, Beyotime, Shanghai, China) at room temperature for 1 h. Finally, enhanced chemiluminescence reagents (K12045-d20, Advansta, CA, USA) were used to detect the protein signals. The Alpha Ease FC gel analysis software (Alpha Innotech, CA, USA) was used to calculate the optical density of protein bands in each group. Primary antibodies were used as below: rabbit anti-mouse NLRP3 (1 : 1000, NBP2-12446, Novus Biologicals, CO, USA), rabbit anti-mouse p20 fragment of Caspase-1 (Caspase-1 p20, 1 : 2000, 89332, Cell signaling technology, MA, USA), rabbit anti-mouse N-terminal fragment of cleaved GSDMD (GSDMD-N, 1 : 500, ER1901-37, HUABIO, Hangzhou, China), and anti-*β* actin (1 : 2000, Proteintech, 20536-1-AP, Wuhan, China).

### 2.8. Real-Time Quantitative Polymerase Chain Reaction (qPCR)

According to the instructions from manufacturer, total RNA was extracted from NSCs using RNAiso Plus reagent kits (9108, TaKaRa Bio, Dalian, China). RNA reverse transcription was performed using PrimeScript™ RT reagent Kit with gDNA Eraser (Perfect Real Time) reagent kits (RR047A, TaKaRa Bio, Dalian, China), and RNA quantitive detection was performed using TB Green® Premix Ex Taq™ II (Tli RNaseH Plus) reagent kit (RR820A, TaKaRa Bio, Dalian, China) in a real time-PCR system (CFX96, Bio-Rad, CA, USA). The tested mRNA included lncRNA-H19, miR-423-5p, NLRP3, and endogenous reference gene glyceraldehyde 3-phosphate dehydrogenase (GAPDH). Primer sequences were synthesized by TaKaRa and listed in Table 1.

### 2.9. Immunofluorescence (IF) Staining

NSCs were fixed with 4% cold paraformaldehyde for 20 min in poly-lysine precoated six-well plates. Then, the cells were washed 3 times in PBS, permeabilized with 0.1% Triton X-100 for 30 min, and blocked by 1% bovine serum albumin for 1 h at room temperature. Next, NSCs were incubated with appropriate primary antibodies: rabbit anti-mouse NLRP3 (1 : 500, NBP2-12446, Novus Biologicals, CO, USA) and rat anti-mouse Nestin (1 : 200, Santa Cruz Biotechnology, CA, USA). Following 3 times washes in PBS, NSCs were incubated with relevant secondary antibodies for 2 h at room temperature, including Alexa fluor plus 488-labeled goat anti-rat IgG antibody (1 : 2000, Invitrogen, A48262, OR, USA) and Alexa fluor plus 594-labeled goat anti-rabbit IgG antibody (1 : 2000, Invitrogen, A32754, OR, USA). Finally, cell nuclei were counterstained with 4′,6-diamidino-2-phenylindole (DAPI, 1 *μ*g/ml, D1306, Thermo Fisher, IL, USA) and mounted with glycerin. All manners were done to minimize light exposure during the staining process. Image capture was performed by a laser scanning confocal microscope (FV3000, Olympus, Tokyo, Japan) and analyzed by the ImageJ software.

NSCs for proliferation assay were incubated with BrdU solution (10 *μ*g/mL, B9285, Sigma-Aldrich, MO, USA) prior to OGD exposure, and then, they were stained with rabbit anti-BrdU primary antibody (1 : 500, ab152095, MA, USA) and Alexa fluor plus 594-labeled goat anti-rabbit IgG secondary antibody (1 : 2000, Invitrogen, A32754, OR, USA) at 3 days after HBO treatment. NSCs for differentiation assay were incubated in inducing medium consisting of DMEM/F12 basic medium and 20 *μ*l/ml B27. The new-generated neuron and astrocyte were, respectively, stained with chicken anti-mouse TUJ1 (1 : 1000, AB9354, Sigma-Aldrich, MO, USA) and rat anti-mouse GFAP (1 : 500, 13-0300, Invitrogen, A-11042, OR, USA) at 14 days after HBO treatment. The secondary antibodies include Alexa fluor 594-labeled goat anti-chicken IgG antibody (1 : 1500, Invitrogen, A-11042, OR, USA) and Alexa fluor plus 488-labeled goat anti-rat IgG antibody (1 : 2000, Invitrogen, A48262, OR, USA). The procedures for NSC proliferation and differentiation staining are performed as described above.

### 2.10. Cell Transfection

To downregulate lncRNA-H19 expression in NSCs, lncRNA-H19 siRNA (siRNA-H19) and negative control siRNA (siRNA-NC) were obtained from GenePharma (Shanghai, China) and transfected into cells at the concentration of 50 nM, respectively. To downregulate the level of miR-423-5p in NSCs, miR-423-5p inhibitor or negative control (miR-NC inhibitor) was obtained from GenePharma and transfected into cells at the concentration of 100 nM, respectively. To overexpress lncRNA-H19 in NSCs, recombinant pcDNA3.1 plasmid and lncRNA-H19 (pcDNA-H19) were constructed by GenePharma and transfected into cells at the concentration of 100 nM, and pcDNA3.1 plasmid without lncRNA-H19 (pcDNA-NC) was used as a negative control. To overexpress miR-423-5p in NSCs, miR-423-5p mimic or negative control (miR-NC mimic) was obtained from GenePharma and transfected into cells at the concentration of 50 nM, respectively. NSCs were seeded into six-well plates at the density of 2 × 10^5^ cells/well and cultured for 24 h. Then, the above transfections were performed using Lipofectamine RNAi MAX Reagent (13778075, Invitrogen, OR, USA) in accordance with the protocols from manufacturer. The transfected NSCs were harvested at 48 h later for subsequent experiments.

### 2.11. Double Luciferase Reporter Assay

The potential binding sites between lncRNA-H19 and miR-423-5p were predicted by the online bioinformatics tool LncBase V2 (http://carolina.imis.athena-innovation.gr). The fragment of lncRNA-H19 containing the binding sequence of miR-423-5p was cloned into pmirGlo vector (Promega, WI, USA) and named lncRNA-H19 wild-type vector (H19-WT). The binding sequence of miR-423-5p in lncRNA-H19 was mutated using MutanBest kit (TaKaRa, Dalian, China) and named lncRNA-H19 mutant vector (H19-MUT). NSCs were cotransfected with the lncRNA-H19 vectors (either H19-WT or H19-MUT) and miRNAs (either miR-423-5p or miR-NC) using Lipofectamine 3000 Reagent (L3000008, Invitrogen, OR, USA); the transfected cells were cultured 48 h and collected for luciferase activity measurement in a dual-luciferase reporter assay system (Promega, WI, USA).

The putative binding sites of miR-423-5p to target gene NLRP3 were predicted by online bioinformatics tool microT-CDS (http://www.microrna.gr/microT-CDS). The 3′UTR fragment of NLRP3 containing the predicted binding sites to miR-423-5p was cloned into pmirGlo vector (Promega, WI, USA) and named NLRP3 wild-type vector (NLRP3-WT). Mutation of miR-423-5p binding sites in the 3′UTR of NLRP3 was generated as described above and named NLRP3 mutant vector (NLRP3-MUT). NLRP3 vectors (either NLRP3-WT or NLRP3-MUT) and miRNAs (either miR-423-5p or miR-NC) were cotransfected into NSCs. The luciferase reporter assays were performed as described above.

### 2.12. Scanning Electron Microscopy (SEM)

Following OGD exposure or HBO treatment, adherent NSCs on poly-L-ornithine/fibronectin-coated plates were harvested and washed 3 times in PBS, fixed with 2.5% glutaraldehyde and 1% osmium tetroxide at 4°C for 1 h, respectively. After another 3 times washes in PBS, NSCs were dehydrated in gradient alcohol, dried in liquid carbon dioxide critical point dryer, and coated by gold film. Then, the ultrastructural morphology of pyroptotic NSCs was observed and captured under a scanning electron microscope (Carl Zeiss, Germany).

### 2.13. Statistical Analysis

The results were presented as mean ± SD and analyzed by GraphPad Prism (Version 8.3.1, GraphPad Software, CA, USA). One-way analysis of variance and least significant difference post hoc test were used to calculate the statistical difference. Pearson's correlation coefficient was applied to test for the correlations between lncRNA-H19, miR-423-5p, and NLRP3 expressed in NSCs. *P* < 0.05 was defined as the significant standard.

## 3. Results

### 3.1. HBO Treatment Protected NSCs against OGD-Induced Injury

In order to investigate NSC viability upon HBO treatment after OGD exposure, cells were divided into six groups: unexposed and untreated control group (Con group), OGD exposure group (OGD group), OGD exposure and 1.0 ATA HBO treatment group (OGD+HBO 1.0 ATA group), OGD exposure and 1.5 ATA HBO treatment group (OGD+HBO 1.5 ATA group), OGD exposure and 2.0 ATA HBO treatment group (OGD+HBO 2.0 ATA group), and OGD exposure and 2.5 ATA HBO treatment group (OGD+HBO 2.5 ATA group). Cell viability and LDH level in each group were, respectively, detected by CCK-8 and LDH assay.

**Figure 1:**
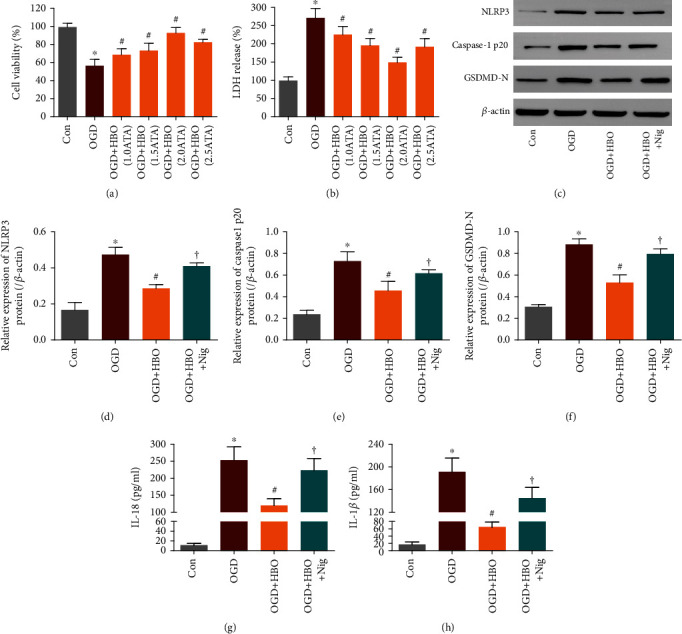
HBO inhibited the activation of NLRP3/Caspase-1/GSDMD signaling in NSCs following OGD. (a, b) NSC viability and release of LDH were tested following OGD exposure and HBO treatment in diverse pressure. (c) The protein expression of NLRP3, Caspase-1 p20, and GSDMD-N in Con, OGD, OGD+HBO, and OGD+HBO+Nig groups was determined by WB. (d–f) Quantitative analysis on the expression of NLRP3, Caspase-1 p20, and GSDMD-N protein in the four groups. (g, h) The level of IL-1*β* and IL-18 produced by NSCs in the four groups was measured by ELISA. ^∗^*P* < 0.05 vs. the Con group. ^#^*P* < 0.05 vs. the OGD group. ^†^*P* < 0.05 vs. the OGD+HBO group.

As shown in Figures 1(a) and 1(b), OGD exposure significantly reduced the viability of NSCs and induced LDH release compared with the Con group. However, HBO treatment rescued the loss of NSC viability and ameliorated cell cytotoxicity in a pressure-dependent manner, especially in the value of 2.0 ATA, indicating that HBO notably protected NSCs against OGD-induced injury. Therefore, in the following experiments, the pressure of 2.0 ATA was employed to investigate the effect of HBO treatment on NSC pyroptosis and neurogenesis after OGD.

### 3.2. HBO Inhibited the Activation of NLRP3/Caspase-1/GSDMD Signaling in NSCs after OGD Exposure

To evaluate the response of canonical pyroptosis signaling in NSCs to OGD exposure and HBO treatment, cells were divided into four groups: Con group, OGD group, OGD exposure and 2.0 ATA HBO treatment group (OGD+HBO group), and OGD exposure and 2.0 ATA HBO combined with NLRP3 agonist nigericin treatment group (OGD+HBO+Nig group). Expression of crucial molecules in the pathway, namely, NLRP3, Caspase-1 p20, and GSDMD-N, were determined by WB. IL-1*β* and IL-18 released from pyroptotic cells were examined by ELISA.

As presented in Figures 1(c)–1(h), compared with the Con group, the level of NLRP3, Caspase-1 p20, and GSDMD-N protein was significantly increased, along with a substantial release of IL-1*β* and IL-18 in the OGD group. Nevertheless, HBO treatment prevented the upregulation of NLRP3, Caspase-1 p20, and GSDMD-N, as well as the production of inflammatory cytokines in the OGD+HBO group. Further study showed that the inhibitory effect of HBO on pyroptosis signaling was abrogated by NLRP3 agonist administration in the OGD+HBO+Nig group. These data suggested that HBO treatment inhibited the activation of NLRP3/Caspase-1/GSDMD pathway induced by OGD in NSCs.

### 3.3. HBO Mitigated NLRP3-Dependent Pyroptosis of NSCs Triggered by OGD

To confirm the inhibitory effect of HBO treatment on NSC pyroptosis following OGD, NLRP3/Nestin colabeling immunofluorescence (IF) staining was carried out and the expression of NLRP3 was analyzed using a laser confocal microscope. Experimental grouping was the same as that in the previous section.

**Figure 2:**
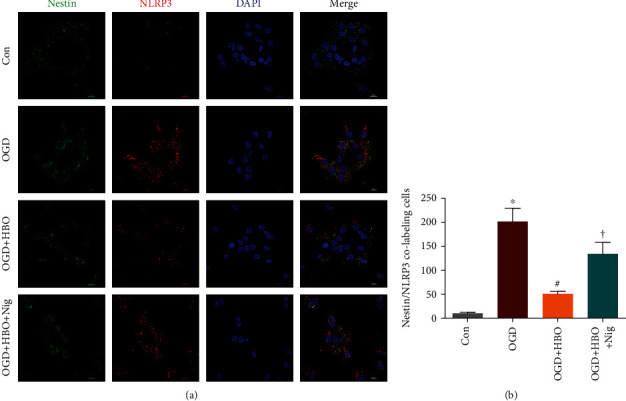
HBO mitigated NLRP3-dependent pyroptosis of NSCs caused by OGD. (a) Representative immunofluorescence staining of NLRP3 and Nestin in NSCs of Con, OGD, OGD+HBO, and OGD+HBO+Nig groups. Nestin^+^: green; NLRP3^+^: red; DAPI^+^: blue. Scale bar = 10 *μ*m. (b) Quantitative counting of NLRP3/Nestin colabeling cells in the four groups. ^∗^*P* < 0.05 vs. the Con group. ^#^*P* < 0.05 vs. the OGD group. ^†^*P* < 0.05 vs. the OGD+HBO group.

As stated in Figure 2(a), NLRP3 positive staining (red) and Nestin positive staining (green) in the cytoplasm of NSCs were observed in each group. Quantitative counting (Figure 2(b)) showed that NLRP3/Nestin colabeling cells in OGD group were evidently increased compared to the Con group, while treatment with HBO resulted in a decrease of colabeling cells in the OGD+HBO group. However, compared with the OGD+HBO group, the population of colabeling cells significantly increased in the OGD+HBO+Nig group. These data indicated that OGD-induced NLRP3 elevation in NSCs was inhibited by HBO treatment.

**Figure 3:**
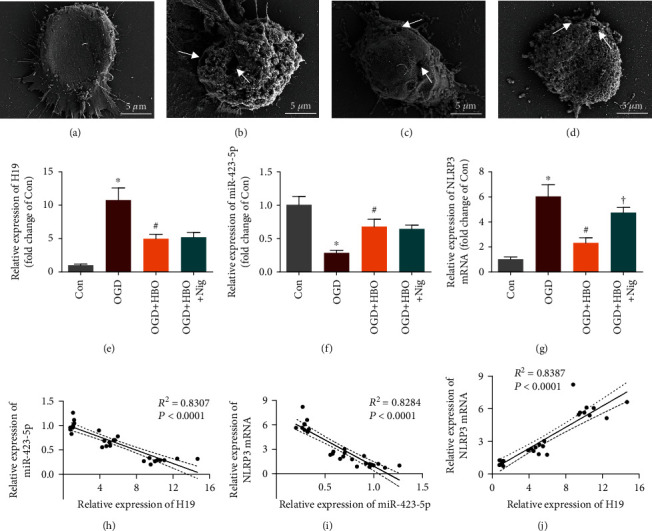
Expression of lncRNA-H19, miR-423-5p, and NLRP3 in NSCs exposed to OGD was altered by HBO treatment. (a–d) Representative scanning electron microphotographs of pyroptotic NSCs in Con, OGD, OGD+HBO, and OGD+HBO+Nig groups. White arrow: GSDMD pores. Scale bar = 5 *μ*m. (e–g) Statistical analysis on the expression of lncRNA-H19, miR-423-5p, and NLRP3 mRNA in the four groups detected by qPCR. ^∗^*P* < 0.05 vs. the Con group. ^#^*P* < 0.05 vs. the OGD group. ^†^*P* < 0.05 vs. the OGD+HBO group. (h) Pearson's analysis on the correlation between lncRNA-H19 and miR-423-5p. (i) Pearson's analysis on the correlation between miR-423-5p and NLRP3 mRNA. (j) Pearson's analysis on the correlation between lncRNA-H19 and NLRP3 mRNA.

Anatomically, the morphological change of pyroptotic NSCs under a scanning electron microscope was observed. As shown in Figures 3(a)–3(d), NSCs in the Con group exhibited smooth and intact plasma membrane, round cell body, and normal protrusions. NSCs in the OGD group displayed typical pyroptosis signs: a plenty of GSDMD pores on plasma membrane and a damaged integrity, pyroptotic body formation, osmotically swelled cell, and an eventual rupture like a fried egg. However, compared with the OGD group, GSDMD pores on plasma membrane were significantly reduced, the damage of membranal integrity was attenuated, and cellular morphology tended to be normal in NSCs of the OGD+HBO group. Additionally, NSC incubation with nigericin resulted in an increase of GSDMD pores on plasma membrane, aggravation of cell swollen, and finally ruptured in the OGD+HBO+Nig group. Taken together, the data revealed that OGD-induced NSC pyroptosis was mitigated by HBO treatment via targeting NLRP3.

### 3.4. HBO Altered the Expression of lncRNA-H19, miR-423-5p, and NLRP3 in NSCs following OGD

In order to investigate how HBO treatment regulated NLRP3-dependent pyroptosis in NSCs following OGD, qPCR was applied to determine the expression of lncRNA-H19, miR-423-5p, and NLRP3 mRNA in Con, OGD, and OGD+HBO groups.

As presented in Figures 3(e)–3(g), the level of lncRNA-H19 and NLRP3 mRNA in the OGD group was markedly higher than that in the Con group, but upregulation of the two molecules was reversed by HBO treatment in the OGD+HBO group. The expression of miR-423-5p in the OGD group was much lower than that in the Con group, but the downregulation was reversed by HBO treatment in the OGD+HBO group. Additionally, the level of lncRNA-H19 and miR-423-5p in the OGD+HBO+Nig group had no statistical difference compared with the OGD+HBO group, but the NLRP3 mRNA expression was significantly increased. Moreover, Pearson's correlation analysis showed a positive correlation of the expression between lncRNA-H19 and NLRP3 mRNA, but they were negatively correlated with miR-423-5p (Figures 3(h) and 3(i)). Taken together, the data indicated that HBO treatment inhibited lncRNA-H19 and NLRP3 mRNA expression but augmented the level of miR-423-5p in NSCs exposed to OGD.

### 3.5. lncRNA-H19 Acted as a Molecular Sponge of miR-423-5p to Target NLRP3

Following bioinformatic analysis and prediction, the binding of lncRNA-H19, miR-423-5p, and NLRP3 was confirmed by dual-luciferase reporter assay. siRNA-NC, siRNA-H19, pcDNA-NC, pcDNA-H19, miR-NC mimic, miR-423-5p mimic, miR-NC inhibitor, and miR-423-5p inhibitor were, respectively, transfected into NSCs to downregulate and overexpress lncRNA-H19 or miR-423-5p in corresponding groups. The regulatory effects were determined by qPCR and WB.

**Figure 4:**
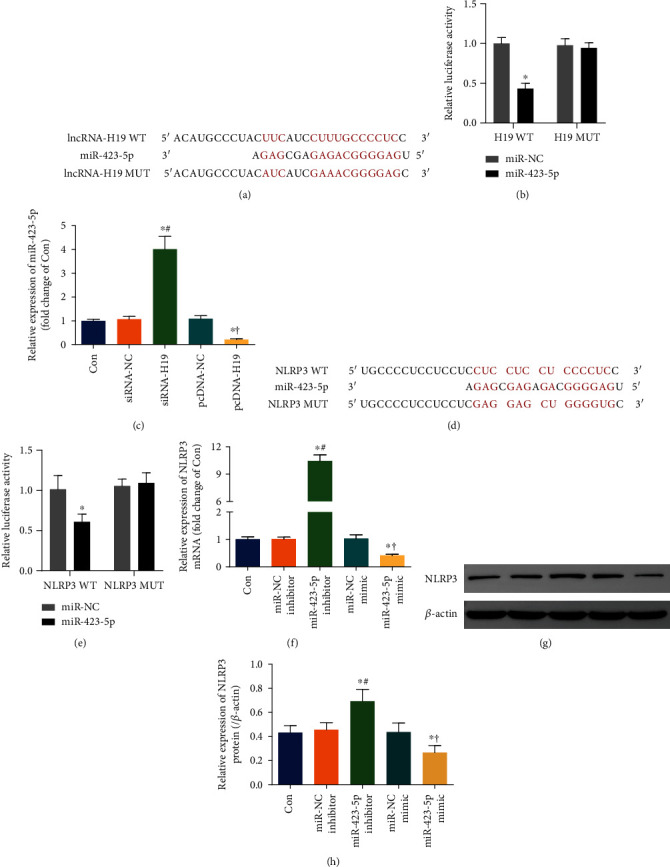
lncRNA-H19 functioned as a molecular sponge of miR-423-5p to target NLRP3. (a) The binding site between miR-423-5p and wild-type lncRNA-H19 (H19-WT) predicted by bioinformational tool LncBase V2; the mutant lncRNA-H19 (H19-MUT) sequence was shown as well. (b) Double luciferase assay was performed to verify the binding of lncRNA-H19 and miR-423-5p. ^∗^*P* < 0.05 vs. the negative control group (miR-NC). (c) Expression of miR-423-5p in NSCs transfected with either siRNA-H19 or pcDNA-H19 or corresponding negative control (siRNA-NC or pcDNA-NC) was examined by qPCR. ^∗^*P* < 0.05 vs. the Con group; ^#^*P* < 0.05 vs. the siRNA-NC group; ^†^*P* < 0.05 vs. the pcDNA-NC group. (d) The predicted binding site between miR-423-5p and the 3′UTR of wild-type NLRP3 (NLRP3-WT) obtained from bioinformational tool microT-CDS; the mutant sequence of NLRP3 (NLRP3-MUT) was also shown. (e) The binding of miR-423-5p and NLRP3 was examined by double luciferase assay. ^∗^*P* < 0.05 vs. the miR-NC group. (f) Expression of NLRP3 mRNA in NSCs transfected with either miR-423-5p inhibitor or miR-423-5p mimic or corresponding negative control (miR-NC inhibitor or miR-NC mimic) was detected by qPCR. ^∗^*P* < 0.05 vs. the Con group; ^#^*P* < 0.05 vs. the miR-NC inhibitor group; ^†^*P* < 0.05 vs. the miR-NC mimic group. (g, h) WB determination and statistical analysis of NLRP3 protein in NSCs transfected as above. ^∗^*P* < 0.05 vs. the Con group; ^#^*P* < 0.05 vs. the miR-NC inhibitor group; ^†^*P* < 0.05 vs. the miR-NC mimic group.

The predicted and mutant binding sequence of lncRNA-H19 and miR-423-5p is presented in Figure 4(a). Dual-luciferase reporter assay revealed that miR-423-5p attenuated the luciferase activity of H19-WT, but not of H19-MUT. Additionally, miR-NC was found to influence neither H19-WT nor H19-MUT luciferase activity (Figure 4(b)). The qPCR assay showed that downregulation of lncRNA-H19 induced an elevation of miR-423-5p, and overexpression of lncRNA-H19 caused an obvious decline of miR-423-5p in NSCs (Figure 4(c)). The data suggested that lncRNA-H19 was available to bind with miR-423-5p and negatively affect its expression.

The predicted and mutant target sites of miR-423-5p and NLRP3 are presented in Figure 4(d). miR-423-5p mitigated the luciferase activity of NLRP3-WT, but that of NLRP3-MUT was not affected. In addition, NLRP3-WT and NLRP3-MUT luciferase activity did not show any change in the presence of miR-NC (Figure 4(e)). The qPCR and WB analysis stated that the level of NLRP3 mRNA and protein was boosted by miR-423-5p inhibitor and was suppressed by miR-423-5p mimic (Figures 4(f) and 4(g)). The data indicated that NLRP3 was the target of miR-423-5p, and its expression was negatively regulated by miR-423-5p.

Based on the molecular sponge property of lncRNA [15] and the above results, it could be speculated that lncRNA-H19 positively regulated the expression of NLRP3 via acting as a molecular sponge to inhibit miR-423-5p in NSCs.

### 3.6. lncRNA-H19/miR-423-5p/NLRP3 Axis Was Involved in NSC Pyroptosis following OGD

To further validate whether lncRNA-H19/miR-423-5p/NLRP3 axis was responsible for NSC pyroptosis induced by OGD, cells were divided into six groups: Con group, OGD group, siRNA-NC transfection and OGD exposure group (OGD+siRNA-NC group), siRNA-H19 transfection and OGD exposure group (OGD+siRNA-H19 group), siRNA-H19 and miR-NC inhibitor cotransfection and OGD exposure group (OGD+siRNA-H19+miR-NC inhibitor group), and siRNA-H19 and miR-423-5p inhibitor cotransfection and OGD exposure group (OGD+siRNA-H19+miR-423-5p inhibitor group). Expression of NLRP3 mRNA and protein in each group was, respectively, determined by qPCR and WB. The level of IL-1*β* and IL-18 produced from pyroptotic NSCs was detected by ELISA.

**Figure 5:**
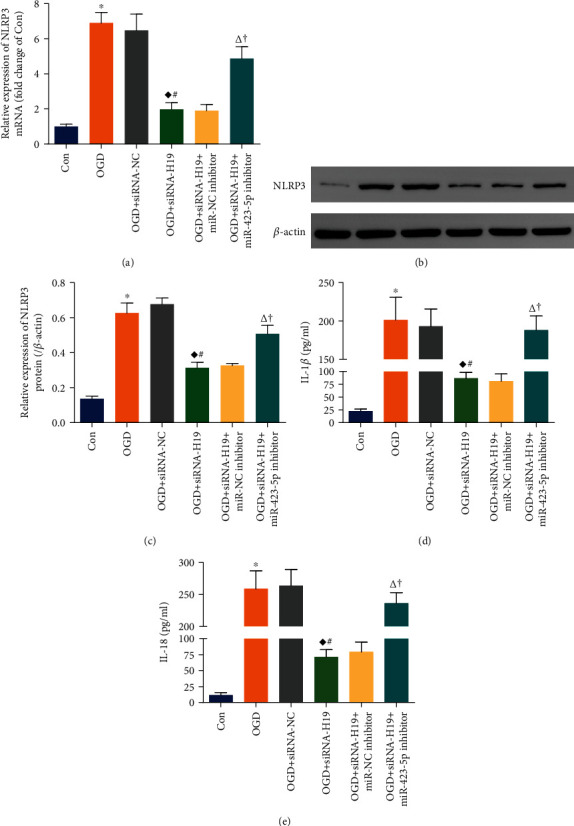
lncRNA-H19/miR-423-5p/NLRP3 axis was involved in NSC pyroptosis following OGD. (a) NSCs transfected with either siRNA-NC or siRNA-H19 or siRNA-H19+miR-NC inhibitor or siRNA-H19+miR-423-5p inhibitor were exposed to OGD and followed by qPCR assay of NLRP3 mRNA. (b, c) WB detection and statistical analysis on the expression of NLRP3 protein in NSCs treated as above. (d, e) ELISA was performed to examine IL-1*β* and IL-18 released from NSCs which were treated with the same manners as above. ^∗^*P* < 0.05 vs. the Con group; ^♦^*P* < 0.05 vs. the OGD group; ^#^*P* < 0.05 vs. the OGD+siRNA-NC group; ^∆^*P* < 0.05 vs. the OGD+siRNA-H19 group; ^†^*P* < 0.05 vs. the OGD+siRNA-H19+miR-423-5p inhibitor group.

As stated in Figure 5(a), the level of NLRP3 mRNA in the OGD group was obviously higher than that in the Con group, while the upregulation was eliminated by siRNA-H19 transfection in the OGD+siRNA-H19 group. There was no statistical difference between the OGD group and the OGD+siRNA-NC group. In addition, NLRP3 mRNA expression in the OGD+siRNA-H19+miR-423-5p inhibitor group was substantially increased compared to the OGD+siRNA-H19 group, but no statistical difference was found between the OGD+siRNA-H19 group and the OGD+siRNA-H19+miR-NC inhibitor group. Similarly, the protein level of NLRP3 in the six groups changed with NLRP3 mRNA (Figure 5(b)). Moreover, IL-1*β* and IL-18 also varied in a similar pattern with the expression of NLRP3 (Figures 5(c) and 5(d)).

These data suggested that OGD induced NSC pyroptosis through upregulating lncRNA-H19 to suppress miR-423-5p and thereby increased NLRP3 activity to trigger the canonical pyroptosis cascade. Downregulation of lncRNA-H19 attenuated NLRP3-dependent pyroptosis and the production of inflammatory cytokines, but the effect was abrogated by miR-423-5p inhibitor.

### 3.7. HBO Protected NSCs against Pyroptosis by Inhibiting lncRNA-H19/miR-423-5p/NLRP3 Axis following OGD

To verify whether lncRNA-H19/miR-423-5p/NLRP3 axis was responsible for the protection of HBO against NSC pyroptosis following OGD, cells were divided into six groups: OGD group, OGD+HBO group, pcDNA-NC transfection and OGD exposure and HBO treatment group (OGD+HBO+pcDNA-NC group), pcDNA-H19 transfection and OGD exposure and HBO treatment group (OGD+HBO+pcDNA-H19 group), pcDNA-H19 and miR-NC mimic cotransfection and OGD exposure and HBO treatment group (OGD+HBO+pcDNA-H19+miR-NC mimic group), and pcDNA-H19 and miR-423-5p mimic cotransfection and OGD exposure and HBO treatment group (OGD+HBO+pcDNA-H19+miR-423-5p mimic group). Expression of NLRP3 mRNA was analyzed by qPCR. The protein level of NLRP3, Caspase-1 p20, and GSDMD-N were determined by WB. IL-1*β* and IL-18 were examined by ELISA.

**Figure 6:**
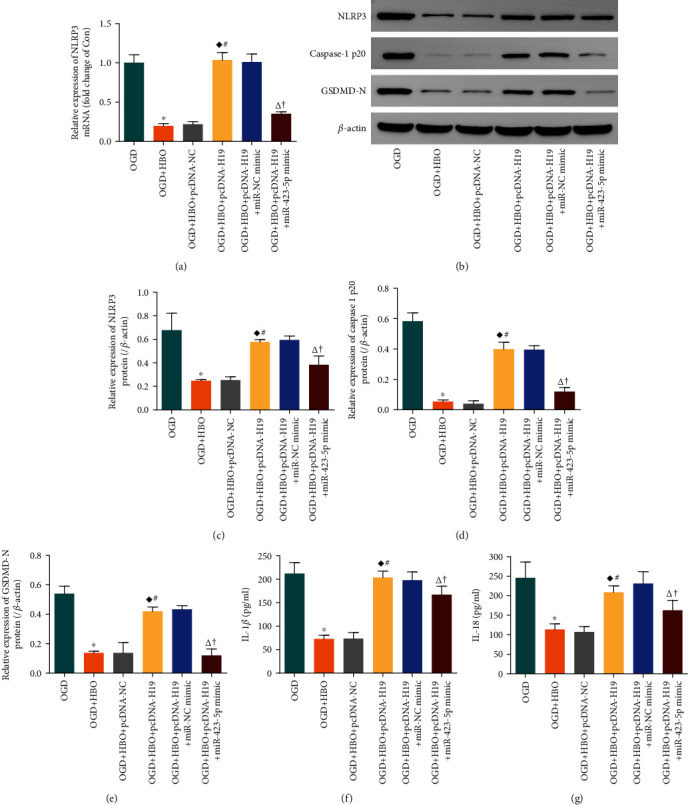
HBO attenuated NSC pyroptosis by inhibiting lncRNA-H19/miR-423-5p/NLRP3 axis following OGD. (a) NSCs transfected with either pcDNA-NC or pcDNA-H19 or pcDNA-H19+miR-NC mimic or pcDNA-H19+miR-423-5p mimic were exposed to OGD and treated by HBO; the level of NLRP3 mRNA was analyzed with qPCR. (b) Expression of NLRP3, Caspase-1 p20, and GSDMD-N protein in NSCs treated as above was determined by WB. (c–e) Statistical analysis on the protein expression of NLRP3, Caspase-1 p20, and GSDMD-N in the above six groups. (f, g) The level of IL-1*β* and IL-18 released from NSCs in the above six groups was examined using ELISA. ^∗^*P* < 0.05 vs. the OGD group; ^♦^*P* < 0.05 vs. the OGD+HBO group; ^#^*P* < 0.05 vs. the OGD+HBO+pcDNA-NC group; ^∆^*P* < 0.05 vs. the OGD+HBO+pcDNA-H19 group; ^†^*P* < 0.05 vs. the OGD+HBO+pcDNA-H19+miR-NC mimic group.

As shown in Figure 6(a), NLRP3 mRNA in the OGD+HBO group was lower than that in the OGD group. Although no statistical difference was observed between the OGD+HBO+pcDNA-NC group and the OGD+HBO group, the level of NLRP3 mRNA in the OGD+HBO+pcDNA-H19 group markedly raised compared to the OGD+HBO group. In addition, there was no statistical difference between the OGD+HBO+pcDNA-H19+miR-NC mimic group and the OGD+HBO+pcDNA-H19 group, but NLRP3 mRNA in the OGD+HBO+pcDNA-H19+miR-423-5p mimic group was seriously decreased compared with the OGD+HBO+pcDNA-H19 group. Moreover, the expression of NLRP3, Caspase-1 p20, and GSDMD-N protein in six groups changed in a similar tendency with NLRP3 mRNA (Figures 6(b)–6(e)). The changes of IL-1*β* and IL-18 were also consistent with NLRP3 in each group (Figures 6(f) and 6(g)).

Accordingly, these data implied that HBO treatment inhibited NSC pyroptosis via suppressing lncRNA-H19/miR-423-5p/NLRP3 axis following OGD. lncRNA-H19 overexpression blocked the inhibition of HBO treatment on NLRP3-dependent pyroptosis signaling in NSCs, but the effect was abolished by overexpression of miR-423-5p.

### 3.8. HBO Restraint of lncRNA-H19-Associated Pyroptosis Benefited NSC Proliferation and Neuronal Differentiation following OGD

To further assess the influence of lncRNA-H19-associated pyroptosis on NSC proliferation and differentiation, cells were divided into five groups: Con group, OGD group, OGD+HBO group, OGD+HBO+pcDNA-H19 group, and OGD exposure and HBO treatment and pcDNA-H19 transfection and NLRP3 antagonist MCC950 administration group (OGD+HBO+pcDNA-H19+MCC950 group). BrdU/DAPI colabeling staining was performed to investigate the proliferation of NSCs at 3 days following HBO treatment. TUJ1/DAPI and GFAP/DAPI colabeling staining were performed, respectively, to investigate the neuronal and astrocytic differentiation of NSCs at 14 days following HBO treatment.

**Figure 7:**
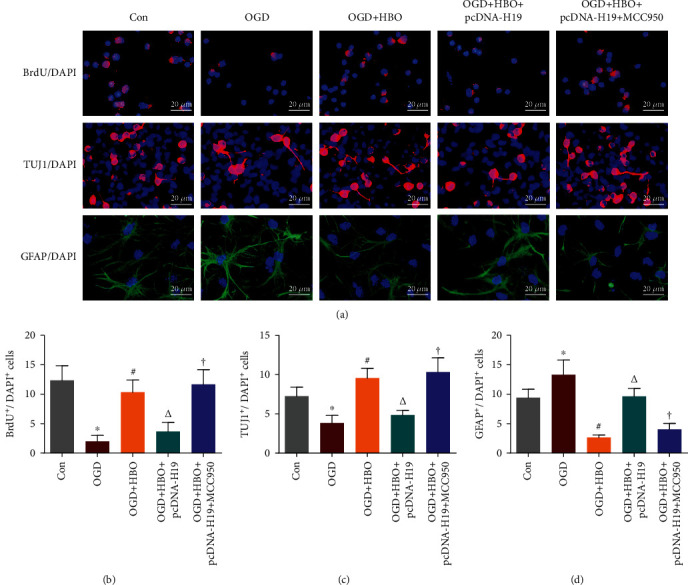
HBO restraint of lncRNA-H19-associated pyroptosis produced a favorable effect on NSC neurogenesis after OGD. (a) BrdU/DAPI colabeling staining was performed to investigate the proliferation of NSCs at 3 days following HBO treatment. TUJ1/DAPI and GFAP/DAPI colabeling staining were, respectively, performed to investigate the neuronal and astrocytic differentiation of NSCs at 14 days following HBO treatment. BrdU^+^: red; TUJ1^+^: red; GFAP^+^: green; DAPI^+^: blue. Scale bar = 20 *μ*m. (b–d) Quantitative counting on the three kinds of colabeling cells in Con, OGD, OGD+HBO, OGD+HBO+pcDNA-H19, and OGD+HBO+pcDNA-H19+MCC950 group. ^∗^*P* < 0.05 vs. the Con group; ^#^*P* < 0.05 vs. the OGD group; ^∆^*P* < 0.05 vs. the OGD+HBO group; ^†^*P* < 0.05 vs. the OGD+HBO+pcDNA-H19 group.

As shown in Figures 7(a)–7(c), compared with the Con group, NSCs exposed to OGD led to a decrease of BrdU/DAPI and TUJ1/DAPI positive cells in the OGD group. However, treatment with HBO significantly increased the number of BrdU/DAPI and TUJ1/DAPI positive cells in the OGD+HBO group compared to the OGD group. Additionally, in comparison to the OGD+HBO group, transfection with pcDNA-H19 caused a reduction of the two kinds of positive cells in the OGD+HBO+pcDNA-H19 group, but the number was substantially increased in the OGD+HBO+pcDNA-H19+MCC950 group. These data implied that HBO treatment rescued the impact of OGD exposure on NSC proliferation and neuronal differentiation through inhibiting lncRNA-H19-associated pyroptosis.

As presented in Figures 7(a) and 7(d), the number of GFAP/DAPI positive cells in the OGD group was more than that in the Con group, whereas a decrease occurred in the OGD+HBO group. In addition, compared with the OGD+HBO group, GFAP/DAPI positive cells were evidently increased in the OGD+HBO+pcDNA-H19 group, but the number was markedly decreased in the OGD+HBO+pcDNA-H19+MCC950 group. These data suggested that HBO treatment prevented the astrocytic differentiation of NSCs following OGD by repressing lncRNA-H19-associated pyroptosis.

## 4. Discussion

It has been documented that the poor capacity of neural regeneration poststroke is predominantly due to the low survival and neuronal differentiation rates of NSCs [2]. HBO treatment is an effective therapy to attenuate hypoxic/ischemic injury and promote neurogenesis after stroke, but the underlying mechanism is not yet fully elucidated. This present study is aimed at investigating the effect and mechanism of HBO treatment on NSC pyroptosis following OGD, as well as its influence on the neurogenesis property of NSCs. Our findings provided the first evidence that OGD triggered NLRP3-dependent pyroptosis of NSCs and led to neurogenesis impairment. HBO treatment alleviated the pyroptotic death of NSCs through lncRNA-H19/miR-423-5p/NLRP3 axis. Moreover, HBO restraint of NSC pyroptosis produced a beneficial effect on NSC proliferation and neuronal differentiation following OGD.

Pyroptosis, a novel form of programmed death, can induce cellular plasm membrane disruption and the release of inflammatory cytokines, which further leads to inflammation in the extracellular environment. The morphological and pathological hallmarks of pyroptosis are quite distinct from other well-known death types of cell death such as apoptosis and necroptosis [23]. Studies have noted that the canonical mechanism of pyroptosis is mediated by NLRP3/Caspase-1/GSDMD signaling [24]. NLRP3 recruits Caspase-1 and other components to assemble inflammasome which serves as an activation complex [25]. The activated Caspase-1 cleaves unmatured pro-IL-1*β* and pro-IL-18 into matured IL-1*β* and IL-18, as well as cleaves the full-length GSDMD into N-terminal and C-terminal fragments [25, 26]. With the translocation and perforation of GSDMD-N on plasma membrane, cell exhibits nucleus condensation, cytoplasmic swelling, membrane rupture, and release of cellular contents, including matured IL-1*β* and IL-18, inducing inflammation [26]. Studies have also shown that pyroptosis plays a vital role in the cell death and inflammation pathogenesis after cerebral hypoxia and ischemia. Targeted modulation of pyroptosis has become a potential strategy to attenuate cell injury and improve outcomes [27, 28].

As noted previously, pyroptosis has been observed in microglia and neuron in central nervous system [29, 30]. However, emerging evidence shows that pyroptosis also occurs in NSCs [31]. The crucial initiator NLRP3 has been viewed as the most promising target to intervene pyroptosis and secondary inflammatory response [32, 33]. Accordingly, whether NLRP3-dependent pyroptosis signaling in NSCs responds to OGD exposure and HBO treatment was investigated in the present study. Our findings suggested that OGD exposure gave rise to the activation of NLRP3/Caspase-1/GSDMD signaling in NSCs, as well as the elevation of inflammatory cytokines IL-1*β* and IL-18. HBO treatment inhibited the activation of pyroptosis signaling in NSCs and inflammatory response elicited by OGD, but the effect was abrogated by NLRP3 agonist nigericin. Therefore, it could be inferred that HBO attenuated the NLRP3-dependent pyroptosis of NSCs following OGD exposure.

lncRNA has been implicated in multiple biological activities by functioning as a molecular decoy to competitively bind miRNA and prevent its inhibitory effect on the target gene [5, 6]. lncRNA-H19, a transcriptional product of the maternally expressed and paternally imprinted gene H19 [34], was initially recognized as an oncogene in the progression of diverse tumors, including glioblastoma, bladder, and gastric cancer [35–37]. Until recently, the multifaceted roles of lncRNA-H19 in cerebral hypoxic/ischemic injury were uncovered and attracted increasing attention. Since the expression of lncRNA-H19 was positively correlated with neurological deficit severity and plasma inflammatory cytokine level following ischemic brain injury, it was viewed as a biomarker to predict the outcome of stroke [38]. It has also been found that the upregulated lncRNA-H19 contributed to neuronal autophagic death and aggravated ischemic injury in the setting of OGD [7]. Inhibition of lncRNA-H19 protected neuron against OGD-induced injury by targeting miR-19a and Id2 [9]. Moreover, downregulation of lncRNA-H19 reduced the cerebral infarct volume and inflammatory response and facilitated hippocampal neurogenesis and repairing process [8, 38]. Importantly, the latest studies revealed that lncRNA-H19 was involved in microglia and cardiomyocyte pyroptosis, respectively, following retinal and myocardial ischemia injury [10–12]. However, the expression and action of lncRNA-H19 in NSC pyroptosis following OGD exposure remain unclear.

Our study revealed that lncRNA-H19 upregulation contributed to NSC pyroptosis by targeting NLRP3 via miR-423-5p following OGD exposure. Based on the bioinformatic analysis and prediction, we first identified that miR-423-5p was one of direct targets of lncRNA-H19 and NLRP3 was one of functional targets of miR-423-5p. Then, using a subset of verification experiments, we observed that lncRNA-H19 was upregulated and miR-423-5p was downregulated in NSCs exposed to OGD, which was accompanied by an activation of NLRP3-dependent pyroptosis signaling. Downregulation of lncRNA-H19 suppressed the activity of NLRP3 and downstream cascade. Moreover, depletion of miR-423-5p abolished the restraint of lncRNA-H19 downregulation on NLRP3-mediated NSC pyroptosis. Collectively, our findings indicated that lncRNA-H19 acted as a molecular decoy to sponge miR-423-5p, leading to an increase of its negatively modulated target NLRP3, which activated the canonical pyroptosis signaling and induced pyroptotic death of NSCs following OGD. Coincidentally, a recent study reported that the interplay between miR-423-5p and NLRP3 was involved in microglia polarization after spinal cord injury, but the upstream regulatory mechanism of miR-423-5p has not been explored [39].

Since lncRNA-H19 expression was found to be tightly correlated with hypoxic inducement in previous study [35], we then investigated whether OGD-induced lncRNA-H19 elevation was altered in the presence of HBO treatment and its effect on NLRP3-dependent pyroptosis signaling in NSCs. HBO is a powerful therapy that boosts oxygen diffusion in hypoxic/ischemic tissue and alleviates secondary injury through targeting noncoding RNA [13, 14, 40]. It has been reported that HBO treatment induces lncRNA-MALAT1 to target miR-92a/KLF2 for angiogenesis after myocardial infarction [21]. HBO treatment suppressed the degeneration of nucleus pulposus cells via miR-107 and miR-573 [19, 20]. In the current study, we found that HBO treatment suppressed the activation of NLRP3-dependent pyroptosis signaling in NSCs following OGD by downregulation of lncRNA-H19. Overexpression of lncRNA-H19 abrogated the inhibitory effect of HBO on NLRP3-dependent pyroptosis. Given lncRNA-H19 served as a molecular decoy to modulate miR-423-5p, we further demonstrated that miR-423-5p mimics eliminated the reverse effect of HBO treatment on lncRNA-H19 overexpression in pyroptotic NSCs. Therefore, it can be concluded that HBO treatment protected NSCs from OGD-induced pyroptosis by inhibiting lncRNA-H19/miR-423-5p/NLRP3 axis to suppress the canonical pyroptotic signaling.

The important role of lncRNA-H19 in NSC pyroptosis prompted us to further study its influence on neurogenesis following OGD exposure and HBO treatment. It was reported that pyroptosis disrupted the survival and neurogenesis of NSCs after Zika virus infection [31]. On the one hand, pyroptotic death leads to a reduction in the amount of NSCs; on the other hand, the inflammatory cytokines (IL-1*β* and IL-18) released from pyroptotic cells are not conducive to the proliferation and differentiation of the surviving NSCs [41]. IL-1*β* is known to be a negative regulator of NSC proliferation in hippocampal neurogenesis and restoration in many neurological disorders [42, 43]. IL-18 not only inhibits NSC self-renewing and differentiation into neurons but also induces newly differentiated neuronal neurite atrophy and cell death [44, 45]. In this study, we found that NSCs exposed in the given OGD condition exhibited a significant decrease of proliferation and neuronal differentiation and an increase of astrocytic differentiation, indicating that the capacity of neurogenesis was decreased. HBO treatment downregulated lncRNA-H19 to inhibit NLRP3-dependent pyroptotic death of NSCs and inflammatory cytokine production, which markedly rescued the neurogenesis impairment caused by OGD. More importantly, through pcDNA-H19 transfection to NSCs, we found that lncRNA-H19 overexpression blocked NSC proliferation and neuronal differentiation elicited by HBO treatment. Therefore, it can be inferred that HBO attenuated lncRNA-H19-associated pyroptosis of NSCs and produced a favorable effect on neurogenesis following OGD.

## 5. Conclusion

In summary, HBO treatment exhibited a protective effect against OGD-induced injury through restraining NSC pyroptosis and promoting neurogenesis. The underlying mechanism was related to inhibition of lncRNA-H19/miR-423-5p/NLRP3 axis and subsequent silence of NLRP3-dependent pyroptosis signaling. The study expanded our understanding to NSC pyroptosis and provided a new clue to develop therapeutic strategy for neural restoration after stroke.

## Figures and Tables

**Table 1:** The sequences of primers used in this study.

Gene	Forward (5′-3′)	Reverse (5′-3′)
lncRNA-H19	GCAGGTAGAGCGAGTAGCTG	TAGAGGCTTGGCTCCAGGAT
miR-423-5p	GGGCAGAGAGCGAGACTTTTC	CAAGCGCGGGGTAGGAA
NLRP3	TACCCAAGGCTGCTATCTGGAG	TGCTTGGATGCTCCTTGACC
GAPDH	CTCAGGAGAGTGTTTCCTCGTC	CCCTGTTGCTGTAGCCGTAT

## Data Availability

The data used to support the findings are available from the corresponding author upon request.
